# A spontaneous depressive pattern in adult female rhesus macaques

**DOI:** 10.1038/srep11267

**Published:** 2015-06-10

**Authors:** Dongdong Qin, Joshua Rizak, Xunxun Chu, Zhifei Li, Shangchuan Yang, Longbao Lü, Lichuan Yang, Qing Yang, Bo Yang, Lei Pan, Yong Yin, Lin Chen, Xiaoli Feng, Xintian Hu

**Affiliations:** 1 Key Laboratory of Animal Models and Human Disease Mechanisms of the Chinese Academy of Sciences & Yunnan Province, Kunming Institute of Zoology, Chinese Academy of Sciences, Kunming, Yunnan, 650223, China; 2 State Key Laboratory of Brain and Cognitive Science, Institute of Biophysics, Chinese Academy of Sciences, Beijing, 100101, China; 3 Kunming Primate Research Center, Chinese Academy of Sciences, Kunming Institute of Zoology, Chinese Academy of Sciences, Kunming, Yunnan, 650223, China; 4 School of Pharmaceutical Science and Yunnan Key Laboratory of Pharmacology for Natural Products, Kunming Medical University, Kunming, 650500, China; 5 Department of Nuclear Medicine, the Second Affiliated Hospital of Kunming Medical University, Kunming, Yunnan, 650101, China; 6 Nuclear Medical Department, the Fourth Affiliated Hospital of Kunming Medical University, Kunming, Yunnan, 650021, China; 7 Department of Rehabilitation Medicine, the Fourth Affiliated Hospital of Kunming Medical University, Kunming, Yunnan, 650021, China; 8 CAS Center for Excellence in Brain Science, Chinese Academy of Sciences, 320 Yue Yang Road, Shanghai, 200031, China

## Abstract

Non-human primates offer unique opportunities to study the development of depression rooted in behavioral and physiological abnormalities. This study observed adult female rhesus macaques within social hierarchies and aimed to characterize the physiological and brain abnormalities accompanying depressive-like behavior. The behaviors of 31 female rhesus macaques from 14 different breeding groups were video recorded, and the footage was analyzed using the focal animal technique. There were 13 monkeys who never displayed huddling behavior (non-huddlers). The remaining 18 monkeys were divided into two groups according the mean time spent in the huddle posture. Four monkeys were designated as high huddlers, whereas the other 14 monkeys were low huddlers. An inverse relationship was discovered between social rank and depression. High huddlers spent more time engaging in physical contact and in close proximity to other monkeys, as well as less time spontaneously and reactively locomoting, than low huddlers and/or non-huddlers. Cortisol levels measured from the hair were elevated significantly in high huddlers compared with low huddlers and non-huddlers, and the measured cortisol levels were specifically higher in high huddlers than subordinate or dominant control monkeys. Regional cerebral blood flow data revealed significant and widespread decreases in high huddlers compared with non-huddlers.

According to the DSM-V (the fifth edition of the Diagnostic and Statistical Manual of Mental Disorders) diagnostic criteria, major depressive disorder is characterized by five (or more) of the following symptoms: depressed mood, loss of interest, weight change, sleep disturbance, psychomotor agitation or retardation, loss of energy, feelings of worthlessness, difficulty concentrating and recurrent thoughts of death. These symptoms must persist for at least two weeks and must include depressed mood and/or loss of interest. Depression can lead to suicide^1^. In addition to psychological symptoms, depressed patients have a greater risk of developing cardiovascular disease^2^ and type 2 diabetes^3^. The World Health Organization (WHO) currently lists depression as the fourth major cause of disability and estimates that it will become the leading cause of disability and the second leading contributor to the global burden of disease by the year 2020^4^. Epidemiological studies have indicated that depression occurs nearly twice as frequently in women than in men. This prevalence has been ascribed to several biological factors, including genetic predisposition, the hormonal changes associated with reproductive function, and hypersensitivity to hormonal changes known to precipitate depression^5^.

To explore the neurobiological basis of depression and to identify more effective treatments, the construction of valid animal models that closely mimic the behavior and physiology of affected humans is essential^6^. Various rodent models of depression have been developed because of the ease of use of rodents, but the rodent brain is phylogenetically distant from the human brain. In contrast, nonhuman primates, especially macaques, exhibit various similarities to humans, suggesting that a model of depression in macaques could provide an important improvement on earlier rodent studies. First, social functioning is known to be closely linked to depression^7^, and select social behaviors of macaques are similar to those of humans. For example, macaques have strict social structures, a dependence on social relationships, an ability to engage in complex cognitive processes and a range of affective expression. In addition, they exhibit a stress response similar to that of humans^8^,^9^. Second, the brain structure of macaques is similar to that of humans^10^. Third, the rearing environment of macaques, in contrast to that of humans, can be tightly controlled and therefore provides an easier platform for collecting quantitative data^8^,^9^, which makes it possible to study the environmental factors contributing to depression in macaques.

Despite these advantages, few macaque studies have been conducted on the etiology of depression. Paul *et al.* found that maternally deprived rhesus macaques displayed a wide range of behavioral abnormalities that could be reversed by chronic antidepressant treatment^11^. Pryce *et al.* constructed a primate model of depression that exhibited “learned helplessness” by depriving marmoset monkey offspring of biological parenting daily^12^. However, none of these studies reported the development of adult depression under normal developmental conditions, as they all depended on external perturbations such as maternal deprivation. Adult depression was modeled for the first time by Shively *et al.* in female cynomolgus monkeys. This model displayed several behavioral and physiological characteristics similar to those of human depression^13^,^14^, and is particularly important because women are more likely to experience clinically significant depression than men^5^.

In humans, psychosocial factors are strongly correlated with depressive symptoms. Perceived stress, lack of personal control or social support, and low socioeconomic status exhibit the strongest associations of all possible factors^15^,^16^,^17^. Murphy and colleagues reported based on a survey that there is an inverse relationship between depression and social status in the human population^18^. A plausible explanation for this finding is that people with low social status experience excessive physical and psychosocial stressors and lack social support and outlets to address the stressors, which contributes to further social stress and may lead to the development of a variety of diseases^19^.

Similarly, macaque societies have an important mechanism of social organization that mimics the experiences of human beings with low socioeconomic status. In macaques, the dominant position is attained through acts of aggression and intimidation, such as open-mouth threats. In this scenario, low-ranking individuals are subject to more attacks by other animals, behave more vigilantly, lack alternative strategies to hierarchy competition, and spend more time alone and less time engaging in affiliative behaviors than their high-ranking counterparts^19^. These traits are associated with higher physiological and psychological stress indices, and the animals become isolated^20^.

Hypercortisolemia is often reported in severe forms of depression, and considerable data have supported that it represents a state of depression^21^. There are also reports of significantly decreased regional cerebral blood flow (rCBF) in the prefrontal cortex^22^,^23^,^24^,^25^,^26^ and temporal regions^27^ of depressed patients, although no single physiological abnormality has been consistently identified to correlate these findings. Despite the lack of physiological indicators, both high cortisol levels and decreased rCBF are correlates of depression.

Findings from previous studies suggest that macaques of low social rank are good candidates for developing depression. The purpose of the present study was to find spontaneous depressive-like behavior in adult female rhesus macaques (*Macaca mulatta*), to clarify the relationship between social rank and depression, and to characterize the physiological (cortisol) and brain changes (rCBF) associated with depressive-like behavior.

## Materials and Methods

### Subjects

In order to ensure the reliability of obtained data, the monkeys were selected according to the following three entry criteria. First, they must be females and are reproductively intact because of our focus on female depression. Second, they should come from breeding colonies, and have lived in their respective social groups for at least 1 year prior to the initial observation to ensure the stability of social hierarchy. Third, none of them were pregnant or had given birth in the last year to avoid the pre-partum or post-partum depression. Therefore, only 31 females met these requirements among the 2,400 monkeys living in the Kunming Primate Research Center of the Chinese Academy of Sciences and were included in the study.

These 31 monkeys ranged from 6 to 19 years old (9.84 ± 3.43 years). They came from 14 different breeding colonies, each containing 2–4 female monkeys and 1 alpha male. Each colony lived in a connected indoor (2.61 × 2.46 × 2.58 m)-outdoor (2.67 × 2.66 × 2.67 m) cage. Monkeys were provided with both physical and feeding enrichment. Two trapezes and two dog toys (such as hollow balls) were placed in each outdoor cage. In addition, feeding enhancements, such as a foraging apparatus, were presented to enrich the animals’ environments. All animals were given commercial monkey biscuits twice a day with tap water *ad libitum* and were fed fruits and vegetables once daily.

The care and treatment of the monkeys was in strict accordance with the guidelines for the National Care and Use of Animals approved by the National Animal Research Authority (P.R. China) and the Institutional Animal Care and Use Committee (IACUC) of the Kunming Institute of Zoology (approval ID SYDW20111230001-1). All possible efforts were made to minimize the monkeys’ suffering. For example, hair samples were taken from the back of the monkey’s neck using an electric-razor (no anesthetics were used). Routine veterinary care was provided by professional keepers and veterinarians. No animals were sacrificed as a result of this study.

### Behavioral data collection and analysis

Behavioral data for all monkeys were collected at the same time of the year (this period was not the breeding period). The focal animal technique^28^ was used to observe one monkey at a time in the cage, and a digital camera fixed on a tripod was set up in front of the colony to record the animal. Before video recording, the monkeys had seven days to become familiar with the observers and cameras. To avoid disturbing the animals during recording, the observers remained as far as possible (at least five meters) from the monkeys’ cage. According to the DSM-V diagnostic criteria for depression, the depressive symptoms must persist for at least two weeks. Therefore, 14 1-hour recordings were collected for each monkey over a period of no less than two weeks, with half collected in the morning (9:00–11:00) and the other half in the afternoon (14:00–16:00).

Conflict behaviors, active behaviors and affiliative behaviors were recorded throughout the experiment using the focal animal technique^28^. The frequency of conflict behavior was recorded^14^. The duration of active behaviors was quantified as seconds per hour and included spontaneous locomotion (defined as any voluntary movement within the cage, including walking, running, jumping and climbing)^29^ and reactive locomotion (defined as locomotion caused by environmental stimuli). The duration of affiliative behaviors, which included sitting together (within another monkey’s arms’ reach or in contact) and alone (outside of another monkey’s arms’ reach), was also quantified as seconds per hour^14^.

### Social rank determination

Based on the outcomes of her agonistic interactions with other group members, each monkey was assigned a rank using the David’s score (DS) method that had been described in detail in previous studies^30^. Briefly, DS for each member, *i*, of a group is calculated with the formula: DS = w + w_2_ − l − l_2_, where w represents the sum of *i*’s *P*_*ij*_ values (*P*_*ij*_ represents the proportion of wins by individual *i* in her interactions with another individual *j*), w_2_ represents the summed w values (weighted by the appropriate *P*_*ij*_ values) of those individuals with which *i* interacted, l represents the sum of *i*’s *P*_*ji*_ values (*P*_*ji*_ represents the proportion of wins by individual *j* in her interactions with another individual *i*) and l_2_ represents the summed l values (weighted by the appropriate *P*_*ji*_ values) of those individuals with which *i* interacted^30^.

To standardize DS scores (DS_S_) across groups, the smallest DS value (usually the largest negative value) in a group was added to its absolute value to create a zero value, and other DS values in the same group were then added to that same value. The new DS values were then divided by the largest new DS value. For each group, this process created DS_S_ values that ranged from 0 to 1, which indicated the lowest rank (DS_S_ = 0) and the highest rank (DS_S_ = 1) in each respective group.

To determine the stability of social hierarchy, the H value was calculated with a previously described equation^31^: H = [12/(n^3^−n)] 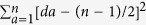, where *da* = 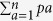, *Pa* refers to the proportion of encounters won by a monkey against another in a pair-wise encounter, and *n* refers to the number of individuals in a group. Calculated H values ranged from 0 to 1, indicating the social hierarchy on a continuum from the least stringent (H = 0) to the most stringent (H = 1).

### Depressive-like behavior

Huddling up was considered representative of behaviors observed in human major depressive disorder because it has been considered the core posture most reflective of depressive-like behavior in macaques^14^,^32^. It is defined as a fetal-like, self-enclosed posture with the head at or below the shoulders during the waking state (i.e., when the monkey had open eyes in the present study), which is accompanied by a relative lack in responsiveness to environmental stimuli that other monkeys attend to.

### Hair sample collection and measurement of cortisol level

Hair samples from all monkeys were collected between 13:30 hours and 15:00 hours. Before the experiment commenced, the hair on the back of each animal’s neck was shaved with an electric razor without the use of anesthetic, with particular attention paid by technicians not to break or damage the skin. After completion of behavioral data collection, newly grown hair was collected as described above. The hair samples were placed into a small pouch of aluminum foil for protection and stored as previously described^33^,^34^.

The hair cortisol extraction procedure has been described in detail in previous studies^33^,^35^. Briefly, hair samples were washed twice in 5 mL of isopropanol (3 minutes each) to remove surface contaminants, dried at 35 °C for 8 hours, and then pulverized using a Retsch ball mill (Retsch M400, Germany) at 26 Hz for 2.5 minutes. Powdered hair (400 mg) was weighed and incubated in 8 mL of methanol at room temperature for 24 hours with slow rotation (230 rpm) to extract the cortisol. The samples were then centrifuged at 8,000 × *g* for 5 minutes, and 4 mL of the supernatant was pipetted into a centrifuge tube and dried under a stream of nitrogen gas. The precipitated extract was reconstituted with 0.5 mL of phosphate-buffered solution (pH: 7.32) and stored at −20 °C until assayed. The cortisol concentration was quantified by radioimmunoassays (RIAs) at the Department of Nuclear Medicine of the Second Affiliated Hospital of Kunming Medical University using a commercially available kit (Cortisol RIA DSL-2000, U.S.A.). The cortisol extraction and RIA analysis were performed in a double-blind manner. Each hair sample was tested three times and the mean of the three hair cortisol values was used to reduce measurement error.

### SPECT scanning

Single photon emission computed tomography (SPECT) was used to measure regional cerebral blood flow (rCBF). SPECT was performed with a dual-head rotating gamma camera (TOSHIBA GCA-7200A, Japan) equipped with a low-energy, high-resolution collimator.

In preparation for the tomography scan, monkeys were given nothing to eat or drink for 4–6 hours prior to the scan. They were then given potassium perchlorate (KClO_4_, 10 mg/kg) to seal off the choroid plexuses. After 30 minutes, atropine (0.05 mg/kg) was injected intramuscularly to prevent the vagal reflex and vomiting. Within 5 minutes, the monkeys were then injected intramuscularly with ketamine (5 mg/kg) and sodium pentobarbital (20 mg/kg). Anesthesia was maintained in accordance with veterinary recommendations, using the lowest possible concentration of sodium pentobarbital to ensure that the macaques were lightly anesthetized. The depth of anesthesia was assessed using physiological parameters (e.g., heart rate and blood pressure, in addition to clinical checks before the scan for muscle relaxation). Anesthetized monkeys were then transferred to a quiet dimly lit room and injected intravenously with technetium-99 m ethyl cysteinate dimer (^99m^Tc-ECD, 2.5 mCi/kg). SPECT scanning was conducted under a double-blind design, and the order of the scans was randomized for each group. The total scanning time for each monkey was 25 minutes. Monkeys were maintained with intermittent positive pressure ventilation to ensure a constant respiration rate during the scan, and the heart rate and blood pressure were monitored throughout the scan. Protocols for animal care, anesthesia, and SPECT were performed under the authority of personal and project licenses granted by the National Animal Research Authority (P.R.China).

Transaxial, coronal, and sagittal slice images were reconstructed by filtered backprojection with a Butterworth filter for an optimum frequency cutoff at 0.20 Hz^36^. In total, 20–22 image slices were reconstructed (thickness of 2.2 mm) for each axis. Regions of interest (ROIs) within the frontal, temporal, parietal, and occipital lobes and the cerebellum were drawn for each slice with reference to the Matsui and Hirano CT brain atlas^37^ by two nuclear medicine physicians.

### Data analysis

Data analysis was conducted using the SPSS software package (SPSS Inc., Chicago, IL, U.S.A.). The normality of the data was determined using the Kolmogorov-Smirnov test. All the data (Kolmogorov-Smirnov tests: all *P*-values > 0.05) except huddling behavior (Kolmogorov-Smirnov test: *P* < 0.00) were normally distributed. The Spearman correlation was used for analyzing the relationship between social rank and huddle behavior. Behavioral and physiological characteristics of depression were analyzed by ANOVA. The alpha level was set at *P* = 0.05, and all *P*-values were generated using two-sided tests. Tukey’s test was used for post hoc comparisons. All data are presented as the mean ± SEM (standard error of the mean).

## Results

### Relationship between social rank and depression

Each monkey was assigned a rank quantized as a given DS_S_ value. The monkeys with a DS_S_ value less than 0.50 were considered subordinate (n = 15), and those with a DS_S_ value equal to or greater than 0.50 were classified as dominant (n = 16). The stability of social hierarchy was measured using the H values, which were all above 0.90 and supported highly strict hierarchies.

Over the course of the experiment, 80% of the subordinate monkeys (n = 12) displayed huddling behavior, whereas only 37.5% (n = 6) of the dominant monkeys displayed huddling behavior. Thus, social rank was inversely associated with depression (correlation between DS_S_ values and time spent in the huddle posture: *rho* = −0.504, *P* = 0.004).

### Behavioral characteristics

**Figure 1:**
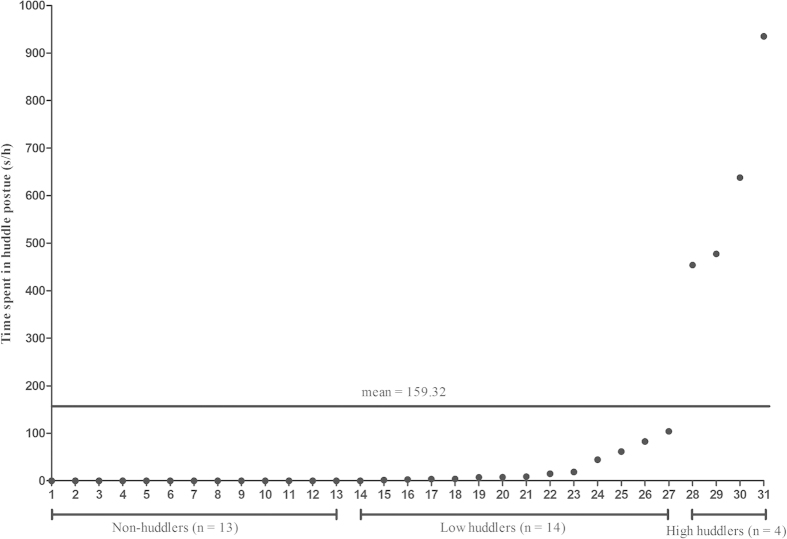
Huddle behaviors. On the X-axis, monkey individuals are divided into three groups: non-huddlers (n = 13), low huddlers (n = 14) and high huddlers (n = 4). The Y-axis displays the mean time spent in a huddle posture (s/h, seconds per hour). Thirteen monkeys never displayed huddling behavior and were designated as non-huddlers. The time spent in the huddle posture was used to divide the remaining 18 monkeys into two groups based on the mean value (mean = 159.32). Four monkeys spent more time in the huddle posture than the mean value and were designated as high huddlers, whereas the other fourteen monkeys were designated low huddlers.

There were 13 monkeys who never displayed huddling behavior (non-huddlers; n = 13). The remaining 18 monkeys were divided into two groups based on mean time spent in the huddle posture. Four monkeys spent more time than the mean value in the huddle posture (mean = 159.32 seconds per hour) and were designated as high huddlers (n = 4), whereas the other monkeys (n = 14) were low huddlers (Fig. 1). Three of the high huddlers were ranked at the bottom with DS_S_ values equal to zero, and the other high huddler was ranked in the middle with a DS_S_ value equal to 0.50.

Behavioral characteristics were compared among high huddlers, low huddlers and non-huddlers. High huddlers spent more time in physical contact and close proximity to other monkeys (1 × 3 groups ANOVA *F*(2, 28) = 4.54, *P* = 0.02; post hoc Tukey’s tests: both *P* < 0.05 ), less time spontaneously locomoting (1 × 3 groups ANOVA *F*(2, 28) = 8.31, *P* = 0.001; post hoc Tukey’s tests: high huddlers versus low huddlers: *P* = 0.32, high huddlers versus non-huddlers: *P* = 0.004), and less time reactively locomoting (1 × 3 groups ANOVA *F*(2, 28) = 3.17, *P* = 0.57; post hoc Tukey’s tests: high huddlers versus low huddlers: *P* = 0.18, high huddlers versus non-huddlers: *P* = 0.05) than low huddlers and non-huddlers (Table 1).

### Basic data

The 31 monkeys were measured with regard to age, body weight (BW) and body mass index (BMI). A statistical analysis indicated a lack of significant differences in age (1 × 3 groups ANOVA *F*(2, 28) = 0.95, *P* = 0.40; post hoc Tukey’s tests: both *P* > 0.05), BW (1 × 3 groups ANOVA *F*(2, 28) = 0.13, *P* = 0.88; post hoc Tukey’s tests: both *P* > 0.05) and BMI (1 × 3 groups ANOVA *F*(2, 28) = 2.69, *P* = 0.09; post hoc Tukey’s tests: both *P* > 0.05) between high huddlers and low huddlers or non-huddlers (Table 1).

### Hair cortisol levels

**Figure 2:**
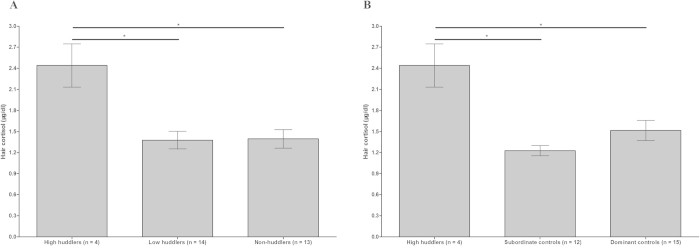
Differences in hair cortisol levels. (**A**) On the X-axis, individuals are divided into high huddlers (n = 4), low huddlers (n = 14) and non-huddlers (n = 13). The Y-axis refers to hair cortisol levels (μg/dl). (**B**) On the X-axis, individuals are divided into high huddlers (n = 4) and subordinate control (n = 12) and dominant control monkeys (n = 15). The Y-axis refers to hair cortisol levels (μg/dl). *P < 0.05. Data are presented as the mean ± SEM.

The levels of cortisol measured from the hair were elevated significantly in high huddlers compared to low huddlers or non-huddlers (Fig. 2A: 1 × 3 groups ANOVA *F*(2, 28) = 8.06, *P* = 0.002; post hoc Tukey’s tests: both *P* < 0.05). There was no significant difference in hair cortisol between subordinates and dominants (1 × 2 groups ANOVA *F*(1, 29) = 0.03, *P* = 0.88), and no significant difference was observed between subordinate and dominant control monkeys (1 × 3 groups ANOVA *F*(2, 28) = 10.01, *P* = 0.001; post hoc Tukey’s tests: *P* = 0.27) when the four high-huddlers were excluded. Nevertheless, the measured cortisol levels were specifically higher in high huddlers than subordinate or dominant control monkeys (Fig. 2B: 1 × 3 groups ANOVA *F*(2, 28) = 10.01, *P* = 0.001; post hoc Tukey’s tests: both *P* < 0.05).

### Regional cerebral blood flow (rCBF)

The monkeys’ rCBF was measured with SPECT scans at the Nuclear Medical Department of the Fourth Affiliated Hospital of Kunming Medical University, and only a few monkeys were allowed to perform the SPECT scans because of limited medical equipment resource. Therefore, 11 monkeys were selected to measure the rCBF, including 4 depressed individuals and 7 normal controls selected randomly from the non-depressed group. The SPECT scans revealed significant differences in rCBF between high huddlers and non-huddlers (Table 2). A decrease in rCBF was found in the cerebral hemispheres of high huddlers compared with non-huddlers (*F*(1, 9) = 6.37, *P* = 0.03). Specifically, the rCBF in high huddlers was significantly decreased in the cerebellum (*F*(1, 9) = 8.38, *P* = 0.02), occipital lobe (*F*(1, 9) = 12.48, *P* = 0.01), and temporal lobe (*F*(1, 9) = 5.77, *P* = 0.04).

## Discussion

In the present study, we report a spontaneous depressive pattern in adult female rhesus macaques that shares several behavioral and physiological characteristics with human depression, including low levels of activity (e.g., spontaneous and reactive locomotion), elevated levels of cortisol, and decreased rCBF in the major parts of the brain. Our results are similar to those of Shively’s study of cynomolgus macaques^14^: high huddlers spent more time in physical contact with and close proximity to other monkeys than low huddlers or non-huddlers. In contrast to Shively, we observed the inverse of the relationship between social rank and depressive-like behavior under normal conditions, which was also reported in the human population^18^. The levels of cortisol measured from the hair were elevated significantly in high huddlers compared to low huddlers or non-huddlers, and the measured cortisol were higher in high huddlers than subordinate or dominant control monkeys of similar social ranking. In addition, brain function was altered in high huddlers, as characterized by low blood perfusion in major parts of the brain.

Similar to Shively’s study^14^, the high huddlers spent more time in physical contact with and close proximity to other monkeys than low huddlers or non-huddlers. This may be somewhat different from human depression because the depressed patients are always socially withdrawn. However, this is advantageous to the depressed monkeys observed in this study. First, depressed monkeys were found to be relatively inactive and they can get warm by keeping close to other bodies^14^. Second, depressed monkeys were less responsive to environmental stimuli, and the vigilance of another monkey close by may make them respond to stimuli that they overlooked or disregarded^14^. Thus, physical contact may be purposeful behavior that the depressed monkeys adopted to ensure survival.

Social defeat can be considered a form of chronic stress in subordinate animals and can provoke behavioral and neuroendocrine changes correlated with a negative emotional state characteristic of human depressive symptoms^38^. It is interesting that there was an inverse relationship between social rank and depression in the present study, as it has been reported in humans that the prevalence of depression is significantly and persistently higher in populations with “low” socioeconomic status than at other levels of socioeconomic status^18^.

Previous studies utilized plasma, saliva or urine to measure cortisol levels. These methods were useful for assaying basal cortisol levels, but the cortisol levels only reflected short-term stress occurring over hours to days and could not assess chronic stress levels occurring over a period of weeks to months without repeated sampling of the animals^39^,^40^. In contrast, cortisol levels measured from hair can reflect chronic stress, as shown in previous rhesus macaque studies^31^,^33^,^35^,^41^. In this study, cortisol levels were measured from hair grown during the behavioral sampling period and were therefore likely to represent the mean cortisol production associated with the chronic stress that the animals experienced. To our knowledge, this is the first study using measurements of cortisol from hair samples to evaluate the function of the HPA axis in depressed animals. The hair cortisol levels were elevated in high huddlers when compared to low huddlers or non-huddlers. There was no significant difference in hair cortisol between subordinates and dominants, similar to our previous findings that cortisol levels were not related to social rank in groups with highly strict hierarchies^31^. When the four high-huddlers were excluded, a significant difference was also not observed between subordinate and dominant control monkeys. Nevertheless, the measured cortisol levels were specifically higher in high huddlers than subordinate or dominant control monkeys of the same social rank. This suggested hypercortisolism was depression specific rather than influenced by the social rank. However, whether hypercortisolism is a cause or effect of depression is a matter of dispute.

SPECT has revealed hypoperfusion in the prefrontal cortex^22^,^23^,^24^,^25^,^26^ and temporal lobes^27^ in patients with depression. This hypoperfusion is positively correlated with depressive symptoms^42^. However, to our knowledge, there have been no reports determining cerebral blood flow in an animal model of depression. Brain SPECT with perfusion agents was performed in this study on anesthetized monkeys. Monkeys designated as high huddlers were found to display hypoperfusion in the whole brain. Some brain areas of high huddlers, including the cerebellum, occipital lobes and temporal areas, were shown to have a significant decrease in the rCBF compared to the same regions in non-huddlers.

The depressive-like symptoms identified in female rhesus monkeys in this study provide a model to help identify environmental factors causing depression and to track physiological and brain changes associated with the development of depression. Nonetheless, the results presented here must be considered with caution because of the relatively small sample size. Further studies are needed to confirm the findings and clarify the extent to which depressive-like symptoms exhibited by female monkeys are reflective of human depression. Whether the abnormalities detailed above reflect the primary pathophysiological changes that produce depression or are secondary responses to alterations of behavior, or adaptations to chronic stress, needs to be further elucidated.

## Additional Information

**How to cite this article**: Qin, D. *et al.* A spontaneous depressive pattern in adult female rhesus macaques. *Sci. Rep.* **5**, 11267; doi: 10.1038/srep11267 (2015).

## Figures and Tables

**Table 1:** **Basic data and behavioral characteristics of high huddlers vs. low huddlers and non-huddlers. Data are presented as the mean ± SEM**.

	**High huddlers (n = 4)**	**Low huddlers (n = 14)**	**Non-huddlers (n = 13)**	***F* (d.f.)**	***P*≤**
Age (years)	12.00 ± 2.89	9.71 ± 0.67	9.31 ± 0.98	0.95 (2,28)	0.40
Body weight (kg)	5.91 ± 053	6.09 ± 0.26	6.18 ± 0.23	0.13 (2,28)	0.88
Body mass index (kg/m^2^)	21.36 ± 1.17	19.53 ± 0.51	21.23 ± 0.60	2.69 (2,28)	0.09
Time spontaneously locomoting (s/h)	46.34 ± 9.53	143.85 ± 23.27	283.22 ± 42.36	8.31 (2,28)	0.001
Time reactively locomoting (s/h)	3.23 ± 1.09	46.71 ± 12.90	63.98 ± 11.26	3.17 (2,28)	0.06
Time Alone (s/h)	1988.57 ± 436.75	1782.01 ± 169.90	1898.93 ± 107.98	0.26 (2,28)	0.78
Time close and in phtsical contact (s/h)	1474.39 ± 606.90	651.04 ± 111.95	588.53 ± 87.69	4.54 (2,28)	0.02

**Table 2:** **Regional cerebral blood flow of high huddlers vs. non-huddlers. Data are presented as the mean ± SEM.**

	**High huddlers (n = 4)**	**Non-huddlers (n = 7)**	***F* (d.f.)**	***P*≤**
Cerebral hemisphere (ROI counts)	210.51 ± 13.24	282.13 ± 19.73	6.37 (1,9)	0.03
Cerebellum (ROI counts)	215.69 ± 11.22	290.75 ± 18.19	8.38 (1,9)	0.02
Occipital lobe (ROI counts)	231.78 ± 13.07	336.72 ± 20.79	12.48 (1,9)	0.01
Temporal lobe (ROI counts)	195.90 ± 12.53	266.93 ± 20.79	5.77 (1,9)	0.04
Parietal lobe (ROI counts)	219.45 ± 12.12	271.26 ± 20.32	3.22 (1,9)	0.11
Frontal lobe (ROI counts)	189.74 ± 20.11	244.97 ± 20.85	3.02 (1,9)	0.12

## References

[b1] Jeon H. J. Depression and suicide. J Korean Med Assoc 54, 370–375 (2011).

[b2] Hare D. L., Toukhsati S. R., Johansson P. & Jaarsma T. Depression and cardiovascular disease: a clinical review. Eur Heart J 35, 1365–1372 (2014). 24282187 10.1093/eurheartj/eht462

[b3] Knol M. J. *et al.* Depression as a risk factor for the onset of type 2 diabetes mellitus. A meta-analysis. Diabetologia 49, 837–845 (2006). 16520921 10.1007/s00125-006-0159-x

[b4] Mathers C. D. & Loncar D. Projections of global mortality and burden of disease from 2002 to 2030. PLoS medicine 3, e442–e442 (2006). 17132052 10.1371/journal.pmed.0030442 PMC1664601

[b5] Garde K. Depression–gender differences. Ugeskr Laeger 169, 2422–2425 (2007). 17594834

[b6] Kalin N. H. Studying non-human primates: a gateway to understanding anxiety disorders. Psychopharmacol Bull 38 Suppl 1, 8–13 (2004). 15278012

[b7] Hirschfeld R. *et al.* Social functioning in depression: A review. Journal of Clinical Psychiatry 64, 268–275 (2000). 10830147 10.4088/jcp.v61n0405

[b8] Barr C. S. *et al.* The utility of the non-human primate; model for studying gene by environment interactions in behavioral research. Genes Brain Behav 2, 336–340 (2003). 14653305 10.1046/j.1601-1848.2003.00051.x

[b9] Kalin N. H. & Shelton S. E. Nonhuman primate models to study anxiety, emotion regulation, and psychopathology. Ann N Y Acad Sci 1008, 189–200 (2003). 14998885 10.1196/annals.1301.021

[b10] Neubert F.-X., Mars, Rogier B., Thomas, Adam G., Sallet, J. & Rushworth, Matthew F. S. Comparison of Human Ventral Frontal Cortex Areas for Cognitive Control and Language with Areas in Monkey Frontal Cortex. Neuron 81, 700–713 (2014). 24485097 10.1016/j.neuron.2013.11.012

[b11] Paul I. A., English J. A. & Halaris A. Sucrose and quinine intake by maternally-deprived and control rhesus monkeys. Behav Brain Res 112, 127–134 (2000). 10862943 10.1016/s0166-4328(00)00173-x

[b12] Pryce C. R., Dettling A. C., Spengler M., Schnell C. R. & Feldon J. Deprivation of parenting disrupts development of homeostatic and reward systems in marmoset monkey offspring. Biol Psychiatry 56, 72–79 (2004). 15231438 10.1016/j.biopsych.2004.05.002

[b13] Shively C. A., Laber-Laird K. & Anton R. F. Behavior and physiology of social stress and depression in female cynomolgus monkeys. Biol Psychiatry 41, 871–882 (1997). 9099414 10.1016/S0006-3223(96)00185-0

[b14] Shively C. A. *et al.* Social stress-associated depression in adult female cynomolgus monkeys (Macaca fascicularis). Biol Psychol 69, 67–84 (2005). 15740826 10.1016/j.biopsycho.2004.11.006

[b15] Simmons L. A., Braun B., Charnigo R., Havens J. R. & Wright D. W. Depression and poverty among rural women: a relationship of social causation or social selection? J. Rural Health 24, 292–298 (2008). 18643807 10.1111/j.1748-0361.2008.00171.x

[b16] Fleischer N. L., Fernald L. C. & Hubbard A. E. Depressive symptoms in low-income women in rural Mexico. Epidemiology 18, 678–685 (2007). 18049184 10.1097/EDE.0b013e3181567fc5

[b17] Adler N. *et al.* Social status and health: a comparison of British civil servants in Whitehall-II with European- and African-Americans in CARDIA. Soc Sci Med 66, 1034–1045 (2008). 18180089 10.1016/j.socscimed.2007.11.031

[b18] Murphy J. M. *et al.* Depression and anxiety in relation to social status. A prospective epidemiologic study. Arch Gen Psychiatry 48, 223–229 (1991). 1996918 10.1001/archpsyc.1991.01810270035004

[b19] Sapolsky R. M. Social status and health in humans and other animals. Annual Review of Anthropology 33, 393–418 (2004).

[b20] Sapolsky R. M. The influence of social hierarchy on primate health. Science 308, 648–652 (2005). 15860617 10.1126/science.1106477

[b21] Gillespie C. F. & Nemeroff C. B. Hypercortisolemia and depression. Psychosom Med 67 Suppl 1, S26–28 (2005). 15953796 10.1097/01.psy.0000163456.22154.d2

[b22] Drevets W. Neuroimaging studies of mood disorders. Biological psychiatry 48, 813–829 (2000). 11063977 10.1016/s0006-3223(00)01020-9

[b23] Holthoff V. *et al.* Changes in brain metabolism associated with remission in unipolar major depression. Acta Psychiatrica Scandinavica 110, 184–194 (2004). 15283738 10.1111/j.1600-0447.2004.00351.x

[b24] Kennedy S. *et al.* Changes in regional brain glucose metabolism measured with positron emission tomography after paroxetine treatment of major depression. American Journal of Psychiatry 158, 899–905 (2001). 11384897 10.1176/appi.ajp.158.6.899

[b25] Kimbrell T. *et al.* Regional cerebral glucose utilization in patients with a range of severities of unipolar depression. Biological psychiatry 51, 237–252 (2002). 11839367 10.1016/s0006-3223(01)01216-1

[b26] Saxena S. *et al.* Differential cerebral metabolic changes with paroxetine treatment of obsessive-compulsive disorder vs major depression. Archives of General Psychiatry 59, 250–261 (2002). 11879163 10.1001/archpsyc.59.3.250

[b27] Yazici K. *et al.* Assessment of changes in regional cerebral blood flow in patients with major depression using the 99m Tc-HMPAO single photon emission tomography method. European Journal of Nuclear Medicine and Molecular Imaging 19, 1038–1043 (1992). 10.1007/BF00180865 1464356

[b28] Altmann J. Observational study of behavior: sampling methods. Behaviour 49, 227–267 (1974). 4597405 10.1163/156853974x00534

[b29] Rogers J., Shelton S. E., Shelledy W., Garcia R. & Kalin N. H. Genetic influences on behavioral inhibition and anxiety in juvenile rhesus macaques. Genes Brain Behav 7, 463–469 (2008). 18045243 10.1111/j.1601-183X.2007.00381.x PMC2785008

[b30] Gammell M. P., De Vries H., Jennings D. J., Carlin C. M. & Hayden T. J. David’s score: a more appropriate dominance ranking method than Clutton-Brock *et al.*'s index. Animal Behaviour 66, 601–605 (2003).

[b31] Qin D. D. *et al.* Social rank and cortisol among female rhesus macaques (Macaca mulatta). Zool. Res. 34, E42–49 (2013). 23572366 10.3724/SP.J.1141.2013.E02E42

[b32] Harlow H. F. & Suomi S. J. Production of depressive behaviors in young monkeys. J Autism Dev Disord 1, 246–255 (1971). 10.1007/BF01557346 5004644

[b33] Davenport M. D., Tiefenbacher S., Lutz C. K., Novak M. A. & Meyer J. S. Analysis of endogenous cortisol concentrations in the hair of rhesus macaques. Gen Comp Endocrinol 147, 255–261 (2006). 16483573 10.1016/j.ygcen.2006.01.005

[b34] Wennig R. Potential problems with the interpretation of hair analysis results. Forensic science international 107, 5–12 (2000). 10689559 10.1016/s0379-0738(99)00146-2

[b35] Feng X. *et al.* Maternal separation produces lasting changes in cortisol and behavior in rhesus monkeys. Proc Nat Acad Sci USA 108, 14312–14317 (2011). 21844333 10.1073/pnas.1010943108 PMC3161556

[b36] Herman G. T. Image Reconstruction From Projections. Real-Time Imaging 1, 3–18 (1995).

[b37] Rizzo G. *et al.* An elastic computerized brain atlas for the analysis of clinical PET/SPET data. European Journal of Nuclear Medicine and Molecular Imaging 22, 1313–1318 (1995). 10.1007/BF00801619 8575483

[b38] Arregi A., Azpiroz A., Fano E. & Garmendia L. Aggressive behavior: Implications of dominance and subordination for the study of mental disorders. Aggression and Violent Behavior 11, 394–413 (2006).

[b39] Keay J. M., Singh J., Gaunt M. C. & Kaur T. Fecal glucocorticoids and their metabolites as indicators of stress in various mammalian species: a literature review. Journal of Zoo and Wildlife Medicine 37, 234–244 (2006). 17319120 10.1638/05-050.1

[b40] Owen M. A., Czekala N. M., Swaisgood R. R., Steinman K. & Lindburg D. G. Seasonal and diurnal dynamics of glucocorticoids and behavior in giant pandas. Ursus 16, 208–221 (2005).

[b41] Qin D. *et al.* Cortisol responses to chronic stress in adult macaques: Moderation by a polymorphism in the serotonin transporter gene. Behav Brain Res 278, 280–285 (2015). 25311283 10.1016/j.bbr.2014.10.001

[b42] Galynker I. *et al.* Hypofrontality and negative symptoms in major depressive disorder. Journal of Nuclear Medicine 39, 608–612 (1998). 9544664

